# Tree Species Traits but Not Diversity Mitigate Stem Breakage in a Subtropical Forest following a Rare and Extreme Ice Storm

**DOI:** 10.1371/journal.pone.0096022

**Published:** 2014-05-30

**Authors:** Karin Nadrowski, Katherina Pietsch, Martin Baruffol, Sabine Both, Jessica Gutknecht, Helge Bruelheide, Heike Heklau, Anja Kahl, Tiemo Kahl, Pascal Niklaus, Wenzel Kröber, Xiaojuan Liu, Xiangcheng Mi, Stefan Michalski, Goddert von Oheimb, Oliver Purschke, Bernhard Schmid, Teng Fang, Erik Welk, Christian Wirth

**Affiliations:** 1 Systematic Botany and Functional Biodiversity, University of Leipzig, Leipzig, Germany; 2 Evolutionary Biology and Environmental Studies University of Zürich, Zürich, Switzerland; 3 Geobotany and Botanical Garden, Martin-Luther-University Halle-Wittenberg, Halle, Germany; 4 Department of Soil Ecology, Helmholtz Centre for Environmental Research - UFZ, Halle, Germany; 5 Waldbau-Institut, University of Freiburg, Freiburg, Germany; 6 State Key Laboratory of Vegetation and Environmental Change, Institute of Botany of the Chinese Academy of Sciences, Beijing, China; 7 Department of Community Ecology, Helmholtz Centre for Environmental Research - UFZ, Halle, Germany; 8 Institute of Ecology, Leuphana University Lüneburg, Lüneburg, Germany; 9 German Centre for Integrative Biodiversity Research (iDiv), Leipzig, Germany; 10 Gutianshan National Nature Reserve, Kaihua, China; Duke University, United States of America

## Abstract

Future climates are likely to include extreme events, which in turn have great impacts on ecological systems. In this study, we investigated possible effects that could mitigate stem breakage caused by a rare and extreme ice storm in a Chinese subtropical forest across a gradient of forest diversity. We used Bayesian modeling to correct stem breakage for tree size and variance components analysis to quantify the influence of taxon, leaf and wood functional traits, and stand level properties on the probability of stem breakage. We show that the taxon explained four times more variance in individual stem breakage than did stand level properties; trees with higher specific leaf area (SLA) were less susceptible to breakage. However, a large part of the variation at the taxon scale remained unexplained, implying that unmeasured or undefined traits could be used to predict damage caused by ice storms. When aggregated at the plot level, functional diversity and wood density increased after the ice storm. We suggest that for the adaption of forest management to climate change, much can still be learned from looking at functional traits at the taxon level.

## Introduction

A widely predicted effect of climate change is an increase in the frequency of extreme weather events [1]. Extreme climate events shape ecological communities and affect plant physiological processes that regulate ecosystem functioning [2], [3]. However, it is still unclear whether aspects of biological diversity can in turn mitigate or influence the impacts of extreme climate events. Systems best suited for the testing of such a hypothesis include those in which a high level of biodiversity coincides with a low probability for extreme events. In this study, we examine how biodiversity affects tree stem breakage caused by an extreme, rare ice storm event in a highly diverse forest in subtropical China at the level of the tree, the taxon and the forest stand. Since experimental studies on impacts of extreme weather events on forests are hardly feasible, we make use of an a-priori scheme for selecting plots that achieves a uniform distribution of diversity similar to comparative experiments.

Forests provide significant local, regional, and global goods and services to society; disturbances in forests are a crucial aspect of ecosystem services, as recovery can be slow due to the longevity of trees. Snow and ice storms can cause large disturbances in forests [4], [5]. Ice load on leaves and branches can cause entire tree stems to break, which can lead to growth reductions and eventually mortality [6]. In addition to ice storm intensity, the extent of damage to trees is likely to depend on properties of the (1) individual tree, such as tree size and form, (2) traits at taxon level, such as wood mechanical properties or leaf habit, or (3) stand level properties, including stand structure, successional age, and stand biodiversity.

Individual tree size can determine the extent of damage caused by ice load [7], [8]. Uprooting has been shown to be more common in small trees and breakage more common in large trees, resulting in a humped-shape curve for the combination of the damage types [9], [10]. At the taxon scale, plant species differ in their susceptibility to damage by a given ice load [11]. While some traits are directly related to susceptibility to damage; i.e. wood density, shearing strength, leaf exposure in winter or leaf surface area, other traits may indirectly affect a species’ susceptibility to damage. Trees have to lift water and nutrients tens of meters to their buds and leaves; their wood construction is constrained by maintaining these functions while at the same time ensuring mechanical stability. Important trade-offs in wood traits relate to the ratio of vessel size to number of vessels and conductivity as well as the construction cost of dense wood and longevity [12], [13]. Trade-offs in leaf traits as described by the leaf economics spectrum (LES) relate the construction costs of leaf tissue structure to liquid phase processes and photosynthetic rates [14]. At the plot scale, damage by ice storms is influenced by topography [7], [11], but also by properties of the forest stand. Stem density [15] and average tree size [7], [16] can increase damage by ice storms. Biodiversity has been shown to stabilize forest functioning in general [17], but may also affect stand properties that increase damage from ice storms. More diverse stands may have higher plant densities [18] and increased crown projection areas [19]. Raindrops have been shown to increase in size in diverse stands [20], so that more diverse canopies may intercept more ice.

Since the focus of ice storm research lies in temperate regions where biodiversity is relatively poor and the probability of ice storms is relatively high, such studies do not usually address biodiversity effects on tree damage [6], [16]. In the present study, we took the opportunity of a rare ice storm event in the biodiversity hotspot of subtropical China [5] to investigate the effects of biodiversity on tree stem breakage and forest functional diversity at the stand level. Southeast China hosts one of the most prominent biodiversity hotspots in the world [21]. Heavy snow and ice storms are extremely uncommon in Chinese subtropical forests and occur with a mean frequency of 50 to 100 years [5]. In January 2008, a series of four severe, long-lasting snow and ice storms afflicted China [5]. These ice storms resulted in the destruction of 17 million ha of forest across China.

In the present study, we investigated the relationship between biodiversity, stem breakage, and uprooting probabilities by this rare ice storm. Our analysis focused on three ecological scales, the individual, the taxon, and the stand scale. We standardized breakage probability for tree size and quantified the magnitude of variation at the taxon and stand level. We examine how functional traits related to the leaf and wood economics spectra affect size corrected individual breakage probabilities at the taxon scale and how biodiversity affects breakage at the stand level. We further analyze tree functional traits and their diversity in forest stands before and after the ice storm. We conclude that individual tree size and functional traits at the taxon scale prevail over attributes at the stand scale in controlling the probability that individual tree stems will break in ice storms.

## Materials and Methods

The study was conducted in the Gutianshan National Nature Reserve, Kaihua County, Zhejiang Province in eastern China (29°8′18″ –29°17′29″ N, 118°2′14″ –118°11′12″ E, 200 m–1280 m asl). The reserve covers an area of 81 km^2^ and had been protected as a National Forest Reserve from 1975 to 2001, after which it was assigned the status of National Nature Reserve (NNR). The forest is representative of Chinese mixed broad-leaved forests. In the Gutianshan NNR, a total of 258 woody species have been recorded [22], with the dominant tree species including *Castanopsis* spp., *Cyclobalanopsis* spp. and *Schima superba* [23].

The climate is characteristic of the subtropics with an annual average temperature of 15.1°C, January minimum temperatures of −6.8°C and July maximum temperatures of 38.1°C. The mean annual precipitation is ∼2000 mm, averaged over 28 years. The highest amount of precipitation occurs in the summer between March and September [23]. There are two peaks of litter fall each year, one in spring (April) and the other in autumn to early winter (between late October and early December) [24]. In January 2008, a series of four severe, long-lasting snow and ice storms affected China [5], including the Gutianshan NNR.

### Study Design, Measurements, and Data Sources

The research permission was provided by the Administration Bureau of the Gutianshan National Natural Reserve, Kaihua, China. No specific permissions were required for our activities. Our field studies did not involve any endangered species. This study is part of a comprehensive project which aims to investigate the effect of biodiversity on the functioning of ecosystems in subtropical forests in China (BEF-China, www.bef-china.de). Datasets of the BEF-China are archived using an instance of the open source BEF-data application software [25] available at http://china.befdata.biow.uni-leipzig.de. In the present study we explicitly cite all data used.

We selected a total of 27 study plots of 30 m×30 m according to a stratified random design using the following criteria: capturing the complete tree diversity and stand age gradient across the NNR in a close-to balanced design and low overall plot damage from the ice storm. Our plot selection procedure achieved a uniform distribution of diversity across stand age similar to that in comparative experiments. Both tree diversity and successional age were later quantified [26]; tree diversity was quantified as species richness based on all woody species with diameter at breast height (DBH) >3 cm, and successional age as age of the 5th largest tree as determined by core drilling of trees from the NNR. Topographic factors largely determine the distribution and mass of ice within a forest [4], [8], [11], [27] and often dominate over biotic effects [8]. We restricted plot selection to the lower damage classes to reduce the bias due to topographic factors. Prior analysis revealed that this procedure was successful: neither slope, exposition, nor the size of the largest broken tree had an effect on tree breakage probabilities; however, trees in higher elevation plots were more likely to break. We therefore included elevation as a covariate in all of the following models (Appendix S1, plot level data: [28]).

### Stand Properties

We considered indicators of vegetation structure and biodiversity as stand level explanatory variables. Components of vegetation structure include the cover of three vegetation layers (upper canopy, lower canopy, and shrub layer), the combined cover of the three layers, and stem density [26], [28]. Components of biodiversity include rarefied species richness, functional diversity (Rao’s quadratic entropy as provided by [29]) and phylogenetic diversity. We performed a principal coordinate analysis to reduce stand properties to two axes that were later used in all statistical models. The first axis scores increased with increasing stem density and total cover, while the second axis scores decreased with increasing functional, phylogenetic, and rarefied diversity (Appendix S1).

### Species Functional Traits

We included wood and leaf functional traits that either have a potential to directly influence the susceptibility to ice breakage or that have the potential to describe general plant strategies. We measured wood density [30] and wood xylem traits [31], [32]. We further used wood mechanical traits provided by the Chinese Research Institute of Wood Industry [33]. For leaf traits, we recorded leaf habit and measured leaf chemical composition [34], [35]. All traits, except for the wood mechanical traits, were measured from trees in the same plots described above. We performed principal coordinate analyses for variable reduction of the selected wood xylem, wood mechanical, and leaf traits, resulting in six main axes (two for each trait type) and a separate seventh variable for wood density (Appendix S2). For the wood xylem traits, the first axis scores increased with the presence of crystals and the presence of paratracheal perenchyma, while the second axis scores decreased with vessel diameter. The first wood mechanical axis scores decreased with shearing, compression, and bending strength, while the second axis scores increased with wood elasticity. The first leaf axis scores increased with specific leaf area and decreased with stomata density; positive scores on the second leaf axis were associated with dentate leaves.

### Tree Individuals

All trees in the study plots were mapped shortly after the ice storm in fall 2008 and early 2009, with broken stems, tree mortality, DBH, and tree height up to the break point being recorded [36]. We used a stratified design to choose which tree individuals were measured in each plot: in the center 10 m×10 m subplot, all trees ≥3 cm DBH were mapped; in the remaining eight subplots only trees ≥10 cm were mapped. Of the 2689 individuals measured, 224 were measured at the base of the stem only. For these individuals, we used major axis regression to interpolate DBH. For the interpolation, we made use of additional data collected from the same individuals in the same plots in the year 2009 (583 tree stems, R^2^ = 0.95).

### Data Analysis

In this study, the main focus was on assessing the impact of biodiversity and species functional traits on individual tree breakage probabilities. Our statistical analysis was based on three approaches necessary to address this focus fully. a) Given that breakage probabilities depend on tree size, and our plots and associated tree species vary in the average size of their trees, we had to standardize the probability of tree breakage with respect to tree size. b) We subsequently compared the standardized probabilities for different plots or species. c) We assessed how much the ice storm actually altered functional diversity and functional trait identity in our study plots. In approaches a) and b) diversity and species traits are factors that mitigate or intensify individual stem breakage. In approach c) the ice storm is the agent changing properties at the forest stand level.

a) Standardizing the probability of tree breakage with respect to tree size.

Our preliminary graphical analysis (Appendix S3) indicated a unimodal form for the probability of stem breakage that depended on tree size. The Power Ricker function offers a unimodal model with a small number of easily interpretable parameters [37]. Fitting a general size-dependent model allowed us to use the deviations from this model for the subsequent analysis comparing tree individuals between plots and species. We used Bayesian inference and Gibbs Sampling via Markov Chain Monte Carlo (rjags: [38]; Appendix S4, Appendix S5) to estimate a DBH dependent breakage probability using the three model parameters 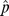, 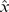 and α:

Stem breakage probability *p_i_* for the *i-th* tree at DBH *x_i_* is given by.
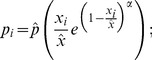
(1)where 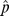 stands for maximum probability of breakage, 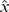 for DBH at maximum probability of breakage, and α for a scaling factor that increases the flatness of the curve at maximum probability of breakage. The larger α, the steeper the slope to maximum mortality and the larger the range of stem DBH values with a high probability of breakage. Incidences of stem breakage *d_i_* come from a Bernoulli distribution, *d_i_ ∼ Bernoulli(p_i_)*, where *p_i_* is given by Equation 1. 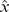 and α were given uninformative lognormal priors and *logit*(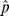) was derived from an uninformative normal distribution.

b) Impact of plot diversity and species traits on standardized stem breakage.

The residuals of the Bayesian model quantify how a given tree stem deviates from the general expectation for its size. In the following, we use the residuals as size standardized increases or decreases of breakage probabilities. We first ranked them using ‘mean rank for ties’ to avoid a bimodal distribution and then scaled them so that negative ranks signify a reduction and positive ranks an increase in breakage probabilities.

We used mixed models to assess the degree to which species and stand properties explain increases or decreases in probability of stem breakage. Taxa and study plots were used as crossed random factors. Preliminary model comparison based on information criteria (AIC) showed that nesting species in families was more parsimonious than using species alone or nesting species in genera or in genera and families. To remove singleton taxa, we used a subset of the data for which we had at least five individuals in each family. Random component models were calculated using Restricted Maximum Likelihood (REML). We quantified the importance of taxa and stand effects using the magnitude of the random effects based on their variance components [39] and used the random effect sizes for species to investigate possible phylogenetic bias after correcting for size and stand effects. Phylogenetic relatedness was quantified using a Maximum Likelihood (ML) method implemented in PhyML (Appendix S6, [40]).

Species and stand properties were treated as fixed effects. For each of the variables we ran four separate models using the subset of the data without missing values. We quantified the relative importance of each variable by calculating the reduction of the variance components at the taxa and stand level from the 1) unconstrained model without the fixed effect to the 2) constrained model including the fixed effect using REML. The relative importance of fixed effects (ω_F_) was calculated analogously to R^2^ values as the ratio of the variance components of the constrained (ω_Rc_) and the unconstrained model (ω_R_). We then re-performed the 3) unconstrained and 4) constrained model using ML to assess the difference in AIC and to perform likelihood ratio tests.

c) Impact of the ice storm on the functional diversity and identity of plots.

The functional identity and diversity before and after the ice storm were calculated based on the basal area of the tree stems. The basal area before the storm was calculated using all the stems, broken or not. To estimate basal area after the ice storm, trees were placed into three potential categories; 1) the basal area of tree individuals unaffected by the ice storm was not changed, 2) the basal area of tree individuals that died due to the ice storm was completely removed from the plot basal area, and 3) the basal area of tree individuals with broken stems or crowns but still living was reduced based on their height [m]/DBH [cm] ratio, to allow for the relative portion of the tree that was damaged. This ratio is large if the height is large relative to the DBH, and it approaches zero if there is only a small part of the stump left from a formerly large tree with large DBH. The ratio ranged between 0 and 1.50, with a median of 0.55. We scaled the ratio to range between 0 and 1 and reduced the basal area of broken but still living trees using this scaled ratio.

We then calculated plot functional identity before and after the ice storm using community weighted means based on functional traits and basal area of each species [41]. All trait axes contained missing values (6% for leaf axes, 30% for the wood xylem axes, and 3% for wood density), with the most missing values from the wood mechanical axes (74%). We therefore excluded the wood mechanical axes from the calculation of plot functional identity and used matrix imputation tools to arrive at a complete trait matrix [42]. Community weighted means for each functional trait axis and functional diversity (Functional Dispersion) were compared before and after the ice storm using t-tests.

Data analysis was performed in the R computing environment [43] using JAGS through rjags [38] for Bayesian modeling and the lme4 package [44] for mixed model analysis (see Appendix S4 and S5 for code used to perform the analysis).

## Results

In the 27 plots 103 tree species were identified, four of which were recorded together in 20 or more plots: *Schima superba* (Theaceae), *Pinus massoniana* (Pinaceae), *Castanopsis eyrei* (Fagaceae), *Myrica rubra* (Myricaceae) and 10 species were recorded together in 10 to 18 plots: *Lithocarpus glaber* (Fagaceae), *Eurya muricata* (Theaceae s.l.), *Albizia kalkora* (Fabaceae), *Quercus serrata* (Fagaceae), *Daphniphyllum oldhamii* (Daphniphyllaceae), *Castanea henryi* (Fagaceae), *Rhododendron ovatum* (Ericaceae), *Toxicodendron succedaneum* (Anacardiaceae), *Loropetalum chinense* (Hamamelidaceae), *Rhododendron latoucheae* (Ericaceae).

**Figure 1:**
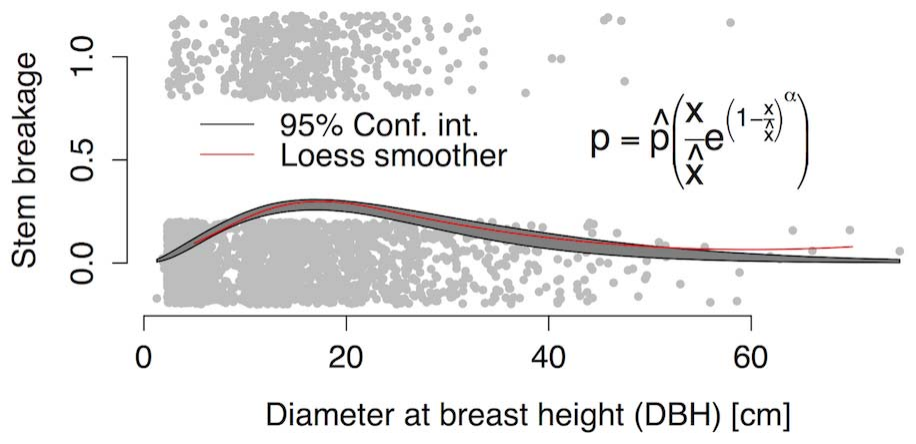
Tree stem breakage after the 2008 ice storm along tree size. Broken stems are scattered around 1 and stems not broken around 0. The red line represents a moving window (Loess) smoother for breakage incidences. The dark grey polygon represents the 95% confidence interval for the probability of breakage of individual stems based on the posterior distribution estimated using a power Ricker function (Equation 1). 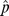 refers to the maximum breakage probability, *x* is the stem DBH, 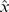 is the DBH at maximum breakage probability and α is a scaling parameter that increases the flatness of the curve at maximum breakage probability.

Of the 2683 stems mapped, 521 broke during the ice storm. In the following we report the mean of the posterior density function and indicate the 2.5% and 97.5% quantiles of the posterior density function in square brackets. Stem breakage probability peaked at a stem DBH 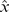 of 17 cm [15 cm; 19 cm]. Maximum probability 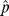 was 0.28 [0.26; 0.31], and the scaling factor increasing the flatness of the curve at maximum breakage probability α was 2.0 [1.6; 2.5] (Fig. 1).

**Figure 2:**
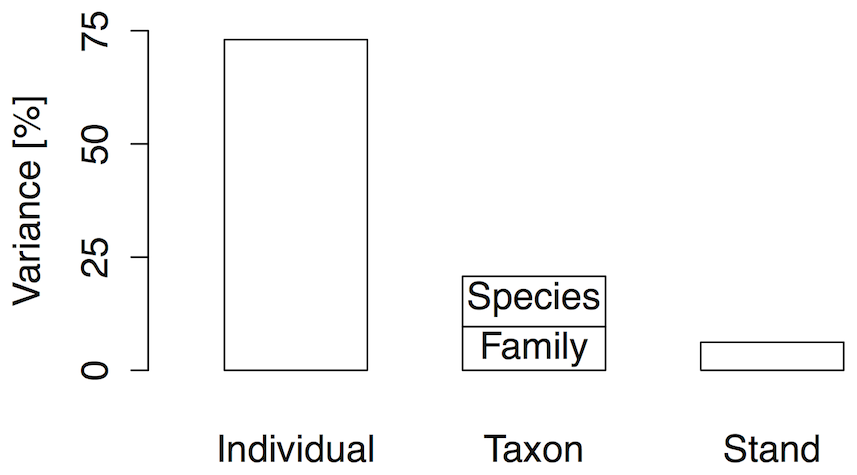
Partitioning the magnitude of variance into the taxon and the stand random variance components. The taxon variance component consisting of species and family effects explained 11% and 10% of the total variance components for DBH corrected breakage probability; the stand variance component explained 6%. The remaining 73% are unexplained variation at the level of the individual tree.

Of the 2683 tree individuals, 2652 were identified to species and represented by at least five individuals in their family. The importance of the taxon given as percent of total variation of the variance components was 10% at the family level and 11% at the species within family level (Fig. 2). Thus, the combined importance of the taxon was more than four times larger than the importance of the stand (5%; Fig. 2). As already indicated by the large variance component related to taxa, the random effects for species (Appendix S4 and S5; data available through [45]) show a clear phylogenetic signal (Blomberg’s K = 0.41, p<0.001; Pagel’s lambda = 0.94, P = <0.001). Among other contrasts, Fagaceae and Fabaceae showed lower breakage susceptibility than Ericaceae and Theaceae (Appendix S6). After correcting for random effects of taxon and stand, model residuals were independent of phylogenetic relatedness (K = 0.09, p = 0.95; lambda <0.001, p = 1).

Stand age reduced the probability of breakage, but neither richness nor the two axes that describe stand properties affected breakage probability (Table 1). Stand age reduced the magnitude of variation at the stand scale by more than a third (ω_F_ = 36%). Of the seven trait axes considered, only the first leaf axis (related to SLA) reduced the probability of stem breakage (Table 1; Appendix S2). The first leaf axis reduced the magnitude of variation at family-scale by ω_F_ = 15% and the variation at species scale by ω_F_ = 4%.

**Table 1:** Fixed effects of stand and species related attributes on the probability of stem breakage from the ice storm.

Fixed effects	Δ AIC	p		dir
Stand properties				
Stand age	−9.13	0.001 **		↓
Richness	1.20	0.371		
Stand axis 1 (e.g. vegetation cover)	−0.30	0.129		
Stand axis 2 (e.g. decreasing functional diversity)	1.66	0.559		
Species traits				
Xylem axis 1 (e.g. crystals)	1.75	0.617		
Xylem axis 2 (e.g. decreasing vessel diameter)	−0.21	0.137		
Mechanics axis 1 (e.g. decreasing shering stress)	1.27	0.393		
Mechanics axis 2 (e.g. wood elasticity)	1.99	0.943		
Wood density	1.25	0.386		
Leaf axis 1 (e.g. SLA)	−3.32	0.021 *		↓
Leaf axis 2 (e.g. dentate leaves)	1.95	0.828		

The column “dir” indicates the direction of the effect, in this case a decrease (↓) with increasing axes scores. Significant differences are indicated as ** at level of <0.01 and * at the level of <0.05.

**Figure 3:**
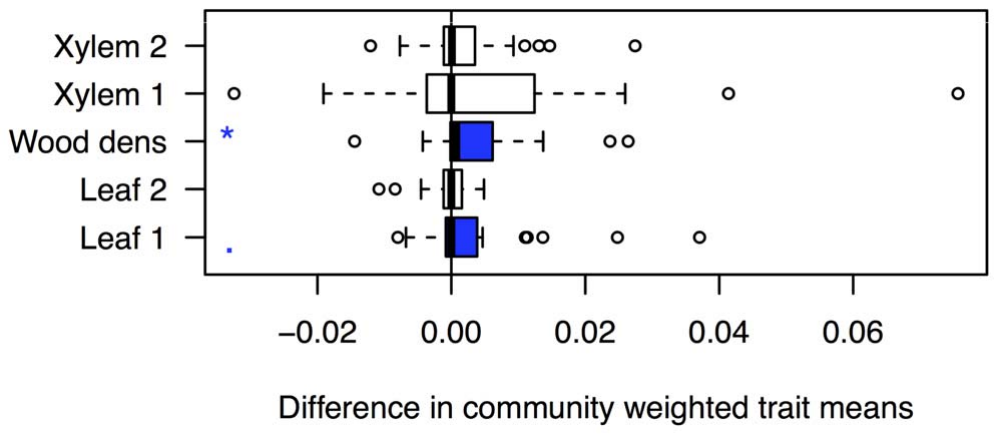
Differences in community weighted mean traits after the ice storm. Blue indicates a significant or marginal increase after the ice storm. Wood density and the first leaf trait axis (increasing SLA) both increased. See Appendix S2 for a detailed description of the species trait axes.

Aggregating plot basal area showed that the ice storm changed functional diversity and functional identity of the study plots. Functional diversity was higher after the ice storm (Functional dispersion, t-test, df = 26, p = 0.046). Plot functional identity also changed, with higher wood density (t-test, df = 26, p = 0.034, Fig. 3) after the ice storm. Additionally, the tendency toward higher SLA values after the ice storm (p = 0.089) reflects our finding that individual trees with higher SLA values have a lower probability of breakage (Table 1).

## Discussion

For the present study, we quantified the effect of tree biodiversity and functional trait identity on the probability of damage following a rare, extreme climate event, an ice storm in South-East China in 2008. From this analysis, we provide evidence that the individual tree size, functional traits of tree species, and the age of the forest stand alter damage probabilities.

We focused on plots with comparably low damage from the ice storm to allow for a more representative evaluation of the wider effects of such events. For example, in the same ice storm and in the same Nature Reserve, Ma et al. show that less than 10% of their study area suffered severe damage [46]. Thus, most of the study area suffered comparably low damage. Our results indicate that ice storms may predominantly affect medium-sized trees (16 – 19 cm DBH) in contrast to larger or smaller ones. Our results differ from other reports on the same ice storm, where a linear increase in damage with DBH was observed (*Cunninghamia lanceolata*: [15]; Bamboo: [8], *Castanopsis* forest: [47]); however, all individuals sampled for the other report had DBH <20 cm and thus belong only to the lower half of the curve we show here (Fig. 1). On the other hand several other studies have demonstrated a similar pattern, where medium-sized trees are most at risk of breakage from ice. Man et al. [7] showed that tree crowns in the size classes of 20 cm – 30 cm broke with highest probability. In another study conducted in a montane evergreen broadleaved forest, trees between 5 and 45 cm DBH were most likely to occur in the two most severe damage classes [48]. Using data from temperate forests, Lafon [10] found that trees with DBH around 18 cm were most likely to suffer severe damage from ice storms, and Cain et al. [9] observed higher rates of stem breakage at DBH values of 15 cm–20 cm in loblolly pines in southeastern Arkansas. This hump-shaped curve is obviously different from the frequently described U-shaped curve for size-dependent mortality [49], suggesting that ice storms may have an effect on the population dynamics of the tree species.

Tools for standardizing responses of individuals at the plot level demonstrate some important advantages for the analysis of biodiversity experiments with trees [50]. In contrast to former experiments in grasslands, trees – unlike grasses and herbs – can be measured more easily at the individual level. However, measurements at the individual level may not be directly comparable between plots, since individuals vary in size or maturity. Additionally, plots in tree experiments are larger than plots in grassland experiments, again increasing heterogeneity of plots. Here we demonstrate how Bayesian models can be used to standardize for the non-linear relationship between breakage probability and stem size, so that breakage can then be compared between plots of differing diversity.

For the goal of assessing the importance of various factors at the scale on which they vary (individual versus species or stand) we found that variance components analysis was an especially useful tool. Variance components quantify the variation due to one factor alone, while mean sums of squares always include the variation of lower levels [39]. For example, the mean squares at the species level of variation include part of the variation at the individual level. In addition, variance components can be expressed in units of the response variable as standard deviations [51], [52]. Working with variance components allowed us to express the importance of a fixed factor, e.g. species traits, relative to the variation of the appropriate random factor, e.g. a taxonomic grouping. In our case, the information at the plant family scale reduced total variation by 10% and the first leaf axis related to SLA reduced this again by 11%. Since plant traits vary at the level of the species, it makes no sense to relate traits to the total variation. Total variation includes the variation of individuals. However, each individual of one species has the same trait value so that trait information cannot explain variability at individual level. If we were to compare the explanatory power of the first trait axis to total variation, we would have to reduce it by one order of magnitude! In this case, we would have to multiply the ratios (11% of 10%). However, since the appropriate scale of variation is the species and not the individual, we can show the importance of the first leaf axis that explains 11% of the variation at the species level. Since plant traits in our study did not vary on the individual scale, because each individual of one species was assigned the same trait value of this species, traits cannot explain any variation at the individual scale and should only be related to the taxon scale.

Here we show that for stem breaking probability, the taxon was more than four times as important as the forest stand (Fig. 2). At the taxon scale, we were surprised to find that only leaf traits affected individual stem breakage probabilities. Trees with higher scores on the first axis of leaf traits, related to increasing SLA among other traits, had a lower probability for stem breakage (Table 1). High SLA values are characteristic of deciduous trees, many of which had dropped their leaves in early winter [24]. Thus, trees without exposed leaves likely had a lower ice load than those with leaves. Damage to deciduous trees was lower across all damage classes, including uprooting, snapping, and breakage, in a study performed after the same ice storm in the same nature reserve [7]. Nevertheless, in our study site both evergreen and deciduous tree species showed a large overlap in SLA values (Bruelheide, unpublished). Low SLA values may also have consequences for twig architecture, regardless of whether the tree has leaves or not, which may affect the amount of ice tolerated by a tree crown.

Over and above the variation explained by the first leaf axis related to SLA, a large part of the variance component in DBH corrected probabilities of breakage at the taxon scale remained unexplained (Fig. 2). This implies that there are unmeasured or undefined functional traits at the taxon scale that could be used to predict the damage caused by ice storms and thus help adapt forest management for climate change.

Our study demonstrates that biotic attributes of stands can mitigate the probability of stem breakage; with increasing stand age stems were less likely to break. Since breakage probabilities were corrected for DBH prior to analysis, this is not a result of increasing mean stem DBH in older stands. Stand age reduced more than a third of the magnitude of variation at the stand scale. Possible mechanisms leading to less susceptible stems in older forests may be linked to the accumulation of biomass with succession. For example, the accumulation of dead wood in older stands of our study sites has been shown to alter soil microbial communities [53], which may feed back on the strength of root anchorage.

However, although our sampling design was optimized to detect the effects of biodiversity, we did not find such an effect on individual stem breakage. In the following we argue that biodiversity may have simultaneous positive and negative effects on stem breakage. Biodiversity may increase stem breakage probability by increasing crown area and asymmetry [19], [54] in addition to increasing stem density [18]. The form of tree crowns may affect susceptibility to ice storm damage [15], [46], and high stand density resulted in in more severe damage in a study by [15]. In a recent study on the same site we have shown that stand biodiversity increases size and variability of raindrops [20]. Diverse forests may similarly offer a diversity of local twig and leaf structures for water to freeze. It is thus possible that stand biodiversity increases the amount of ice captured at canopy level. Diversity may either have no effects on individual stem breakage, or both negative and positive effects, that cancel each other out at the scale of the individual tree. In contrast, at the scale of the stand the ice storm changed the functional identity of the plots (Fig. 3) and thereby increased their functional diversity, reinforcing the argument that disturbances maintain and enhance biodiversity [55].

In conclusion we provide evidence for a general, unimodal size dependency for stem breakage, which has to be standardized to assess effects of the ice storm at plot and species scales. We show that the taxon of a tree individual (family and species) and functional traits affect breakage probabilities far more than do properties of the stand where the tree may be located. While stems with higher SLA were less susceptible, the strong and yet unexplained phylogenetic pattern in breakage probability indicates that much can be learned from looking at functional traits at the taxon level to explain tree damage from ice storms and to help forest management to adapt for climate change.

## Supporting Information

Appendix S1 **Details on the principle coordinate analysis that reduced stand properties to two axes.**
(DOCX)

Appendix S2 **Details on the principle coordinate analysis that reduced wood xylem, wood mechanical, and leaf traits to six main axes.**
(DOCX)

Appendix S3 **Preliminary graphical analysis indicating a unimodal form for the probability of stem breakage dependent on tree size.**
(DOCX)

Appendix S4 **Description of the R script files for the analysis.**
(DOCX)

Appendix S5 **Compressed file containing the analysis scripts in R code.**
(ZIP)

Appendix S6 **Phylogenetic tree and averaged taxon random effect sizes for susceptibility to stem breakage.**
(DOCX)
