# Supplementary material for: Tree Species Traits but Not Diversity Mitigate Stem Breakage in a Subtropical Forest following a Rare and Extreme Ice Storm
Source: PLoS One. 2014 May 30;9(5):e96022. doi: 10.1371/journal.pone.0096022 (PMC4039427; doi:10.1371/journal.pone.0096022)
Supplement: Appendix S1 — Details on the principle coordinate analysis that reduced stand properties to two axes. (DOCX) [file pone.0096022.s001.docx]

Appendix plot_attributes

We used three different types of plot attributes to assess biodiversity effects on ice storm breaking probability: 1) design variables, 2) plot biotic properties apart from the design variables, 3) abiotic properties. We want to control for the influence of abiotic plot properties before we look at biotic properties and thus looked at their impact on deviations from stem breaking probability first. Elevation was the only influential abiotic plot property. It was therefore included as covariate in all subsequent models.

Table 1: Effects of type 3) abiotic plot properties on deviations from stem breaking probability. Likelihood ratio tests comparing two models, one including the property as main fixed effect and one without, both using species within family crossed with plot as random effects.

| Plot attribute | Δ AIC | p (Likelihood ratio) |
| --- | --- | --- |
| largest broken tree | 0.86 | 0.287 |
| northness | 1.61 | 0.534 |
| eastness | 2.00 | 0.840 |
| elevation | -6.16 | 0.004** |
| inclination | 1.24 | 0.382 |

Type 2) biotic variables include forest cover (upper and lower canopy cover, shrub cover, and the incremental sum of all covers), stem density, and rarefied species richness (Bruelheide & Nadrowski 2013), functional diversity (Böhnke 2011), phylogenetic diversity and distinctness (Michalski et al. 2012). We used variable reduction based on Gover distances (Laliberté & Legendre 2010) and Principle Coordinate Analysis to reduce the variables to two main axes explaining 92% of the variation (Mantel test, p < 0.01). The first axis scores increase with total cover and stand density, the second axis scores decrease with rarefied, functional, and phylogenetic diversity. The code for the PcoA can be found in the file scripts/plot_attribute_correlations.r (Appendix scripts).

Figure 1: Principle coordinate analysis of stand properties. (upper tree layer cover: “t_up_cover", lower tree layer cover: "t_low_cover", shrub cover: "shrub_cover", incremental sum of all cover values: "sum_cover", stem density: "density", functional diversity of all leaf and wood traits measured using to Rao’s Q and tree species abundances: “functional_rich”, rarefied richness: “rarefied”, phylogenetic diversity based on the total sum of branch lengths of the phylogenetic tree: “pd_dec_ev”, and phylogenetic distinctness: “mpd_dec_ev”.
