# Supplementary material for: Tree Species Traits but Not Diversity Mitigate Stem Breakage in a Subtropical Forest following a Rare and Extreme Ice Storm
Source: PLoS One. 2014 May 30;9(5):e96022. doi: 10.1371/journal.pone.0096022 (PMC4039427; doi:10.1371/journal.pone.0096022)
Supplement: Appendix S2 — Details on the principle coordinate analysis that reduced wood xylem, wood mechanical, and leaf traits to six main axes. (DOCX) [file pone.0096022.s002.docx]

Appendix species_traits

We used wood and leaf traits to assess biodiversity effects on ice storm breaking probability. Wood traits include xylem traits and traits related to wood mechanic properties. For each of the trait sets we performed a principle coordinate analysis based on a Gover distance matrix (Laliberté & Legendre, 2010). After reducing each trait set to two main coordinates, we used Mantel tests to assess the goodness of fit relative to the original distance matrix.

### Wood: Xylem traits (23 traits)

Wood xylem traits were measured for 96 species based on the IAWA list of wood xylem traits (Böhnke, Eichenberg, & Bruelheide, 2010; Böhnke, Kreißig, Kröber, Fang, & Bruelheide, 2011). They include the distinctness of growth rings ("growth rings"; IAWA 1-2), occurrence of gums and other deposits in hardwood vessels ("deposits”; IAWA 58), fibres with simple bordered pits ("simple pits"; IAWA 61), fibre pits distinctly bordered ("bordered pits"; IAWA 62), fibre pits common in both radial and tangential walls ("pits common"; IAWA 63), fibres with helical thickenings ("fib helic thick”; IAWA 64), axial parenchyma absent or extremely rare ("no axial"; IAWA 75), prismatic crystals present ("crystals": IAWA 136), mean vessel diameter ("vessel diam"), variance coefficient of vessel diameters ("var coef"; IAWA 3-5), number of perforation bars between vessel elements ("perf bars"; IAWA 13-19), vessel ray pits: round (0), both (1), elongated (2) ("ray pits"; IAWA 31-33), helical thickenings: none (0), in part of the cell (1), in whole cell (2) ("ves helic thick"; IAWA 36-39), fibre wall thickness: very thin (1), thin to thick (2), very thick (3) ("fib thick"; IAWA 68 – 70), presence of apotracheal parenchym ("apotrach"; IAWA 76, 77), presence of paratracheal parenchym ("paratrach"; IAWA 78 – 84), both apo- and paratracheal parenchym ("banded", IAWA list 85 – 89), ray width: uniseriate (1), 1-3 cells (2), 4-10 cells (7) ("ray width", IAWA 96 – 98), chrystals in ray cells ("ray crystals", IAWA 37-40), crystals in parenchym cells ("par crystals", IAWA 141, 142), mean pith ray width ("pith ray"), mean length of vascular segments ("segment length"), tylosis type in cells: non, common: sclerotic, common: not sclerotic (“tylos non scl”, “tylos sler”, “no tylosys”, IAWA 56, 57). The first two principle axes explain 85% of the variation in the 23 xylem traits (p < 0.001 ***). The first axis scores increase with presence of chrystals and the presence of paratracheal parenchym, the second axis scores decrease with vessel diameter.

### Wood density

Wood density was measured for nearly all of the species found in our study plots (Nadrowski, Bruelheide, & Böhnke, 2009). However, combining the data with the dataset on wood mechanics was not possible due to missing values, i.e. species for which we knew only wood density, but none of the other wood mechanics traits. We therefore used wood density as separate variable.

### Wood mechanic traits

Wood mechanic traits were digitized for 339 tree species from (Research Institute of Wood Industry; Chinese Academy of Forestry, 1982) and included mean ring width ("ring width"), percent latewood fraction ("late wood"), mean percent shrinkage in radial direction ("shrinkage rad"), mean compression strength parallel to wood grain ("compression par"), mean bending stress ("bending"), modulus of elasticity for bending ("elasticity"), radial shearing strength ("shearing rad"), tangential shearing strength (“shearing tang"), tensile strength parallel to wood grain ("tension"), and wood toughness ("toughness"). Reducing the variables to two principle axes explains 96% of the original variation (p < 0.001, ***). The first wood mechanic axis scores decrease with shearing, compression, and bending strength, the second axis scores increase with wood elasticity.

### Leaf traits

Leaf traits were measured for 129 tree species from our study site (Böhnke, Eichenberg, Kröber, & Bruelheide, 2012; Kröber, Böhnke, Welk, Wirth, & Bruelheide, 2012) and include leaf area ("LA"), specific leaf area ("SLA"), stomata density ("stomata.density"), stomata size ("stomata_size"), a binary leaf thickness indicator ("coriaceous", "papery"), leaf dentation type ("dentate", "pinnate"), leaf longevity (“deciduous”, “evergreen”), as well as nitrogen content ("N"), carbon content ("C"), potassium content ("K"), magnesium content ("Mg"), calcium content ("Ca"), and phosphorous content ("P"). Two principle components explain 89% of the variation (p < 0.01**). The first leaf axis scores increase with specific leaf area and decreases with stomata density; positive scores on the second leaf axis belong to dentate leaves.
