# Supplementary material for: Tree Species Traits but Not Diversity Mitigate Stem Breakage in a Subtropical Forest following a Rare and Extreme Ice Storm
Source: PLoS One. 2014 May 30;9(5):e96022. doi: 10.1371/journal.pone.0096022 (PMC4039427; doi:10.1371/journal.pone.0096022)
Supplement: Appendix S3 — Preliminary graphical analysis indicating a unimodal form for the probability of stem breakage dependent on tree size. (DOCX) [file pone.0096022.s003.docx]

Appendix species_size

The following plots show incidences of stem break for different stem sizes within single families, genera, and species. We show those taxa that have at least 10 occurences in the dataset. We overlay incidences of stem break with a moving average (blue line and grey area) and the general breaking probability function as an outcome of pooling all data from all species (red).

Stem break occurences (black points) for separate families for different stem sizes. Plots include a loess smoother (blue line and grey area) overlaid by the expected breaking probability as estimated based on the Ricker function and data pooled for all taxa.

Stem break occurences (black points) for separate genera for different stem sizes. Plots include a loess smoother (blue line and grey area) overlaid by the expected breaking probability as estimated based on the Ricker function and data pooled for all taxa.

Stem break occurences (black points) for separate species for different stem sizes. Plots include a loess smoother (blue line and grey area) overlaid by the expected breaking probability as estimated based on the Ricker function and data pooled for all taxa.
