# Supplementary material for: Tree Species Traits but Not Diversity Mitigate Stem Breakage in a Subtropical Forest following a Rare and Extreme Ice Storm
Source: PLoS One. 2014 May 30;9(5):e96022. doi: 10.1371/journal.pone.0096022 (PMC4039427; doi:10.1371/journal.pone.0096022)
Supplement: Appendix S4 — Description of the R script files for the analysis. (DOCX) [file pone.0096022.s004.docx]

Appendix script description

In the following we shortly describe the files needed to reproduce our analysis on incidences of stem break in the extreme and rare ice storm in subtropical China.

ice_storm.r: This file performs the main mixed models analysis and links to several other files in the scripts/ folder.

**Files in the scripts/folder**

bayesian_three_parameter.r: R script to run the Bayesian model in the following file.

bayesian_three_parameter_bug.r: Bayesian model to run in the JAGS environment for the three parameter Ricker model.

bayesian_variances.bug.r: Bayesian model to run in the JAGS environment to test decreasing variances for plot to plot distances in functional space after the ice storm.

before_after.r: R script looking at differences after the ice storm. Calculating basal area before and after the ice storm, and looking at functional identity and diversity differences after the ice storm.

compare_unconstrained_constrained.r: R script for comparing nested mixed models. The constrained model adds one fixed effect as additional main effect to the model and AIC, likelihood ratio, as wall as differences in variance components (Δω) are calculated.

extracting_random_species_effects.r: R script that extracts the random species effects from the basic random component model before including additional fixed effects. The script writes the random species effects into a file in the data/ folder.

figure_variance_components.r: R script for producing the figure that shows the relative importance of the variance components of the unconstrained mixed model.

get_varcomp_lmer.r: R script extracting relative variance components (ω) from a mixed model.

phylogeny script.r: R script looking at phylogenetic pattern in the random species effects and in the mixed model residuals after accounting for species.

plot_attribute_correlations.r: R scripts for variable reduction of the plot attributes.

plot_ricker_model.r: R script that produces the figure of incidences of stem break and breaking probabilities for individual stems. Breaking probabilities are derived from the Ricker functions.

plotting_decreasing_variances.r: R script producing the figure of decreasing variances in plot to plot distances in functional space after the ice storm.

prepare_leaf_traits.r: Collecting leaf trait variables from the original data sets.

prepare_plot_attributes.r: Collecting plot attributes from the original data sets.

prepare_wood_traits.r: Collecting wood trait variables from the original data sets.

random_effects_model_selection.r: Model selection of the variance component model before including fixed effects. Candidate models nest taxa differently, such as species in family in class, or species in genera.

significance_omega elevation.r: Collecting the results from the script compare_unconstrained_constrained.r to extract effect size and significance of nested mixed models as well as their variance components. This script accounts for elevation in the unconstrained and constrained model.

significance_omega.r: Collecting the results from the script compare_unconstrained_constrained.r to extract effect size and significance of nested mixed models as well as their variance components.

size_species_interaction.r: Plotting incidences of stem break along stem diameter for separate taxa: families, genera, and species.

trait_correlations.r: Variable reduction for the species traits. Results in eight main components axes, two for each of the wood xylem, wood mechanics, and leaf trait. Plus one wood density variable.
