# Supplementary material for: Tree Species Traits but Not Diversity Mitigate Stem Breakage in a Subtropical Forest following a Rare and Extreme Ice Storm
Source: PLoS One. 2014 May 30;9(5):e96022. doi: 10.1371/journal.pone.0096022 (PMC4039427; doi:10.1371/journal.pone.0096022)
Supplement: Appendix S6 — Phylogenetic tree and averaged taxon random effect sizes for susceptibility to stem breakage. (DOCX) [file pone.0096022.s006.docx]

Appendix phylogenetics :

In our paper “Biodiversity and tree species traits mitigate stem breakage in subtropical forest following a rare and extreme ice storm” we first standardize stem breakage for each tree individual to correct for the effect of individual tree size. After finding a humped shape relationship between breakage and tree size (Figure 1 in the paper), with small trees and large trees suffering more from stem breakage, we use the deviation from this general humped shape curve to quantify an increased or decreased breakage probability for each individual, thus arriving at a quantification for breakage probability that now is independent from individual tree size.

In a second step we partitioned the variance of breakage probabilities into the variance associated to taxa and to site attributes (Figure 2 in the paper), finding that the taxon captured 21 % of the variation, while stand attributes only capture 5 % of the variation. Since our main focus here was the effect of stand biodiversity on breakage probability, we had to make sure that the phylogenetic signal does not interfere with our conclusion on stand biodiversity. For this, there should be no phylogenetic signal in the residuals of the final model testing for stand biodiversity effects. Using the phylogenetic tree below, we quantified the phylogenetic effect on size standardized breakage probabilities. There was a strong phylogenetic signal (Blomberg’s K = 0.41, p < 0.001; Pagel’s lambda = 0.94, P = <0.001). However, including taxon as random effect in the model successfully removed the phylogenetic signal from the model residuals (K = 0.09, p = 0.95; lambda < 0.001, p = 1).

### Phylogenetic tree

The phylogeny for our taxa set was extracted from a larger tree for woody plant species in the Gutianshan National Nature Reserve (Michalski & Durka 2013). In short, for this phylogeny sequence information (matK, rbcL and ITS region) was gathered from GenBank or obtained using barcoding protocols. A maximum likelihood tree was computed and dated using non-parametric rate smoothing and using published fossils as age constraints. For further information, see (Michalski & Durka 2013).

### Taxon random effect sizes

Averaged taxon random effect sizes for susceptibility to stem breakage are provided for the convenience of our readers on the BEF-China project data portal (Nadrowski 2013).

Ericaceae and Theaceae showed higher than expected susceptibility to break in the ice storm, whilst Fagaceae and Fabaceae show lower susceptibility (Figure 1, below).

Figure 1: Phylogenetic tree of the tree species involved in the 2008 ice storm in the Gutianshan National Reserve in subtropical China and average random effect sizes for each taxon. Colors indicate deviations from expected stem breakage probabilities based on individual tree size. Deviation was negative for most of the species, however, e.g. Theaceae and Ericaceae did not deviate as much as e.g. Fagaceae or Fabaceae. Data are available on the data portal of the BEF-China project for the phylogenetic tree (Michalski & Durka 2013) and the random effect sizes for stem breakage probabilities (Nadrowski 2013).

References

Michalski, S.G. & Durka, W. (2013). Phylogenetic tree of woody species in the Gutianshan National Reserve [WWW Document]. *BEF-China data portal*. URL http://china.befdata.biow.uni-leipzig.de/datasets/240

Nadrowski, K. (2013). Deviations from stem breaking probabilities at species level [WWW Document]. *BEF-China data portal*. URL http://china.befdata.biow.uni-leipzig.de/datasets/327
